# Co-similar malware infection patterns as a predictor of future risk

**DOI:** 10.1371/journal.pone.0249273

**Published:** 2021-03-29

**Authors:** Amir Yavneh, Roy Lothan, Dan Yamin

**Affiliations:** 1 Department of Industrial Engineering, Faculty of Engineering, Tel Aviv University, Tel Aviv, Israel; 2 Center for Combatting Pandemics, Tel Aviv University, Tel Aviv, Israel; Politecnico di Torino, ITALY

## Abstract

The internet is flooded with malicious content that can come in various forms and lead to information theft and monetary losses. From the ISP to the browser itself, many security systems act to defend the user from such content. However, most systems have at least one of three major limitations: 1) they are not personalized and do not account for the differences between users, 2) their defense mechanism is reactive and unable to predict upcoming attacks, and 3) they extensively track and use the user’s activity, thereby invading her privacy in the process. We developed a methodological framework to predict future exposure to malicious content. Our framework accounts for three factors–the user’s previous exposure history, her co-similarity to other users based on their previous exposures in a conceptual network, and how the network evolves. Utilizing over 20,000 users’ browsing data, our approach succeeds in achieving accurate results on the infection-prone portion of the population, surpassing common methods, and doing so with as little as 1/1000 of the personal information it requires.

## Introduction

The internet holds an unfathomable amount of malicious content, also known as malware. Malware can come in various forms, such as drive-by-downloads and phishing attacks, which lead to loss of time and money and to personal information theft. During 2015, the FBI received over 280,000 internet cybercrimes complaints, which led to a total loss of over one billion dollars [1]. The statistical office of the European Union further suggested that over 30% of internet users from all over Europe became infected by malware during 2010 [2]. Similar trends were identified by other recent studies [3, 4].

All links in the user’s defense chain affect these risks, from the internet service provider (ISP) to the browser itself. For example, the ISP can block unwanted traffic, and the browser can withhold the user from entering a specific malicious website. Recent studies proposed several advancements to such security components in three directions. First, some researchers examined the differences between users regarding their browsing habits and the corresponding needs for personalized protection [1, 4–11]. Second, several systems addressed the benefit of having a proactive or predictive approach rather than a reactive one; taking action only once the attack occurred could lower the effectiveness of blocking the malware [3, 4, 8, 12–15]. Third, some projects considered the need to maintain a low level of privacy invasion while supplying means of protection to the users [8]. However, most systems still require a great deal of information regarding the user’s activity for their risk evaluation. Even more so, despite these different needs for advancement, all three of them are rarely accounted for together.

The differences between users, both in terms of behavior and personal characteristics (e.g., country and age), govern their risk of becoming infected [1, 4–11]. Ovelgönne et al. [6] collected data from 1.6 million users over eight months and analyzed the binaries installed on the users’ computers to determine their infection risk. They classified users into several categories according to the types and prevalence of their binaries. Their findings suggested that software developers have the highest number of malware compared to the other studied groups, and that users with larger numbers of low-prevalence binaries are at higher risk. Canali et al. [4] showed that users with a higher tendency than the average of entering malicious websites enter more than double the amount of distinct URLs. They also found a strong correlation between visiting pornographic websites and becoming infected with malware, a subject addressed by other studies as well [7, 16]. Furthermore, apart from the categories of the visited websites, patterns in the user’s browsing behavior were also shown as effective for differing between risky users and safe ones. For example, Gratian et al. [8] showed that the average browsing session length and the average time difference between session starts could serve as robust features for classification.

Security systems do not always account for these differences to personalize the protection offered to the users. In some systems, it may be computationally challenging to explicitly consider all aspects of the individual’s behavior to customize her protection. ISP’s standard defense mechanism includes setting a single general protection threshold that distinguishes between malicious URLs and safe ones. This threshold is dynamic, in the sense that it can be adjusted to time-specific threats roaming the internet, but it is identical for all users. This single-threshold-approach suggests that, at any given moment, either safe users are deprived of some harmless content, risky users are allowed access to malicious content, or a combination of both. Accordingly, it is essential to develop an applicable and adjustable mechanism to the user’s personal needs to improve protection.

Due to malware’s dynamic nature and their growing sophistication, protection measures need to be adjusted accordingly. One way to address these changes is to focus on proactive protection. For a security system to be proactive, it needs to predict the level of risk or forecast an upcoming malware attack. The subject of predicting the risk of infection has been addressed in several studies [3, 4, 8, 12–15]. Bilge et al. [13] achieved excellent results in predicting infections by analyzing the binaries installed on users’ computers and identifying ones that are more likely to be attacked. Liu et al. [15] focused on forecasting organization-level cybersecurity incidents. Kang et al. [12] combined behavior-related and malware-related features from their data and epidemic modeling to predict malware infections in different countries. Other studies predicted infections according to wireless traffic data [8], or according to browsing activity [3, 4, 11]. Ben Neria et al. [11] proposed a personalized security framework that focuses on users’ browsing behavior and combines two models: one that evaluates each user’s risk level and another that evaluates the risk level of each web page. Each model utilized the scores produced by the other model iteratively. Canali et al. [4] predicted users’ levels of risk by analyzing their browsing behavior. They used the data from the browsing sessions of 160,000 users and extracted over 70 features to capture each user’s web surfing habits. These features accounted for the time of day in which users were most active, how much time they spent on the internet, and the number of different websites they visited. They used these features with a logistic regression algorithm to predict which users are more likely to be victims of web attacks, with a level of accuracy as high as 87%. A pioneering study by Sharif et al. [3] analyzed HTTP traffic of over 20,000 mobile phone users and survey data collected from part of the users. They analyzed the high-resolution data of users’ past browsing behavior, such as the times of day during which the user browsed and the visited websites’ categories. Using these features, they predicted infections in an upcoming period, with slightly lower accuracy levels than those of Canali et al. [4].

Security habits play a significant part in the practice of online security behavior [17]. As behavior is predominantly affected by habits [18, 19], insight into individuals’ habitual behaviors can predict future actions [20]. However, constant tracking of the users’ activities leads to an invasion of their privacy. Indeed, the studies mentioned above used extensive amounts of personal information or activity tracking, thus extensively invading users’ privacy. Even though the users were anonymized in most studies, their entire activity was recorded and used, suggesting that it can more easily be exploited compared to systems that require less information.

This paper presents an architecture that infers each user’s risk level by considering her historic infection patterns solely, using only a small fraction of her browsing activity data. We answer the need for a personalized threshold for each user and create a model that can predict who will imminently become infected. By focusing only on the users’ previous infection patterns and not their entire browsing activity, we substantially reduce privacy invasion.

## Methods

**Fig 1:**
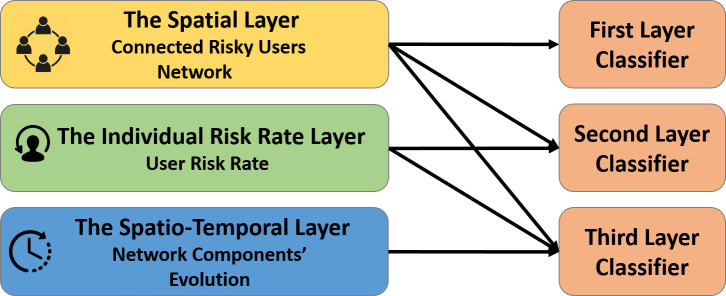
Architecture of the malware prediction framework.

Our malware prediction framework aims to identify users that will be exposed to malware (i.e., enter malicious URLs–hereafter term ’exposed’) during a specific timeframe by analyzing only partial information about the user’s previous exposure history. We consider three modular layers of information in our framework. The first is the *Connected Risky Users Network* layer. This layer includes a conceptual network of users linked to one another if 1) they entered the same domain, and 2) they entered malicious URLs within that same domain. The second layer is the *user risk rate*, which evaluates each user’s rate of exposure. The third is the *Network components’ evolution* layer, which depicts and utilizes the conceptual network’s development throughout time to improve the framework’s prediction power. A schematic diagram of our framework’s architecture is presented in Fig 1. The layers are ordered by their proneness to result in privacy invasion. Namely, first, we considered a first classifier that includes the *Connected Risky Users Network*, then we add the *User risk rate*, and finally, we add information describing the *Network components’ evolution*. In the following sub-sections, we describe in detail each of the three layers comprising our framework.

### The spatial layer–Connected Risky Users Network

The *Connected Risky Users Network* accounts for co-similarities between exposure patterns of risky users. Our underlying assumption is that users with similar previous exposure patterns share a similar level of risk of becoming exposed again [20]. We define risky users as co-similar if they were exposed to malicious content in the same domain. Correspondingly, we create an undirected graph *G*(*V,E,W*), where vertices represent users. We connect any two users *u* and *v*(∈*V*) by an edge *e_u,v_*∈*E* if they entered a malicious URL in the same domain during a past period (see data analysis). The weight of the edge, *w*∈*W*, represents the number of distinct domains in which both users entered a malicious URL. The construction of the network is based *only* on users who entered malicious URLs in the same domains, while ignoring the URL’s identity and how many other (harmless) URLs the users entered in that domain. Moreover, it needs only an anonymized version of the domain name: it suffices to say that two users were exposed to malicious content in some domain *X*, regardless of the domain’s actual name. Hence, the network does not use any personal information. We refer to the users that belong to the network as *Connected Risky Users* (CRUs) and to the network as the *Connected Risky Users Network* (CRUN). Any user with at least one neighbor is included in the network. Some risky users entered malicious URLs in domains where no other user entered a malicious URL. We refer to these users as *Unconnected Risky Users* (UCRUs), and they do not take part in the network. In other words, we leave only nodes (users) with a minimal degree of 1.

### The individual risk rate layer–User Risk Rate

The individual risk rate layer, which we refer to as the *User Risk Rate* (URR), simply evaluates the rate at which users enter malicious websites as an indicator for their future level of risk. For each user *u*, we count the total number of seconds she browsed in the past learning period and the total number of malicious URLs she entered during that time. By dividing the latter by the former, we achieve the user’s rate of entering malicious websites, which we denote by *λ_u_*. Given *λ_u_* and the estimation of the browsing duration, we assume the number of malicious URLs entered by user *u*, denoted by *X_u_*, to be a Poisson random variable. This assumption is consistent with studies that involve identifying malicious events due to user behavior [21, 22], as well as general user browsing behavior [23]. However, we note that the condition of the Poisson distribution that is not necessarily met in our case is the one requiring events (entrances to malicious URLs) to be mutually independent. Thus, to further test our assumption modeled in the URR component of the framework, we conducted a statistical test on the training period, which determined that only 27.2% of the risky users entered malicious websites during that period at a significantly non-Poisson rate (see S1 Text. Statistical analysis for the URR). Therefore, the Poisson distribution seems to serve as a sufficiently good proxy for future exposure.

Essentially, the URR is a single feature that evaluates the probability that the user will enter at least one malicious URL during the prediction period, *t*. Therefore, we define the URR feature for user *u* with a maximal likelihood estimation of the user risk rate ${\hat{\lambda }}_{u}$ from the learning period as follows:

$$URR(u)=P(X_{i}>0|{\hat{\lambda }}_{u})=1-P(X_{u}=0)=1-e^{-{\hat{\lambda }}_{u}t}.$$

### The Spatio-temporal layer–Network component’s evolution

We aimed to gain additional insights from the relations between the user and the network evolvement. The underlying assumption of this part is taken from previous network models of infectious diseases that suggested that earlier infections and changes in centrality measures increase the risk for future infections [24].

**Fig 2:**
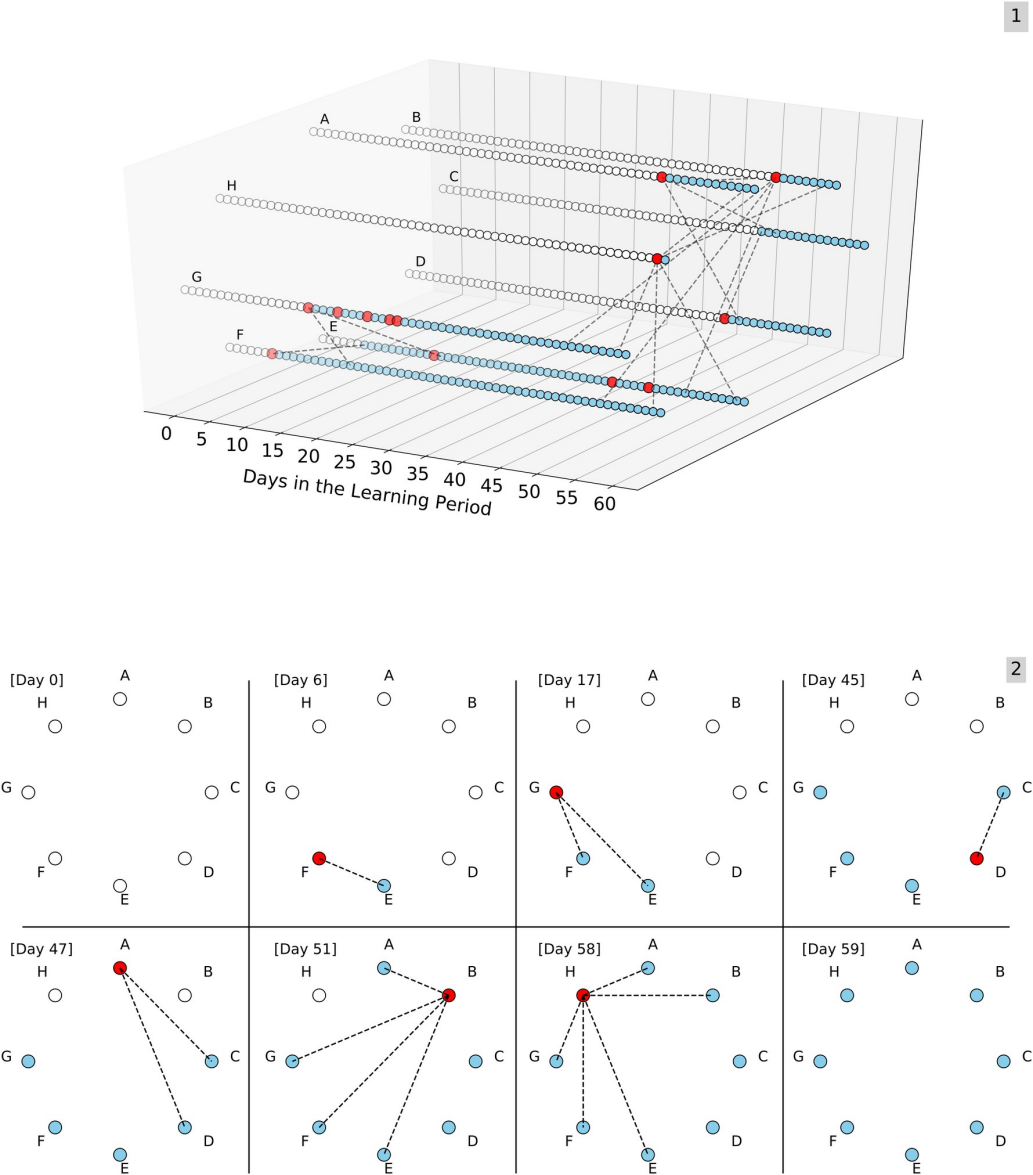
The Spatio-temporal layer. **[1]** An illustrative example of the evolution of one of the connected components in the CRUN during the 60-day learning period. Each of the connected component’s eight users in the example (denoted by nodes A-H) is represented by a vector of circles; a white circle indicates that the user did not belong to the connected component by that day. A blue circle indicates that the node belonged to the network but was not exposed during that day, and a red circle indicates that the user had already been part of the network and was also exposed on the given day. Exposures that occurred before joining the network were not accounted for in this model. **[2]** Eight snapshots depicting the evolution of the connected component, including a snapshot of the first and last days in the learning period, and a snapshot for every day in which edges were added to the connected component. For a gif containing an animation of this process, see S1 Gif.

Thus, for users who at some point belonged to the network during the learning period, we explicitly tracked 1) the first day they became part of the network (i.e., part of the CRUN), and 2) after being part of the network, the day(s) in which they were exposed to malicious URLs (Fig 2).

## Dataset and analysis

### Data and preprocessing

We use web browsing data collected from over 100,000 users worldwide over the course of three months. The data consist of HTTP logs provided by a leading American toolbar company. With the installation of the software, all users agreed that the browsing data, including the order of URLs and the time spent in each one, will be recorded and used for research. The software anonymized the users’ identities, and each user was assigned a new unique ID.

The dataset toolbar company requested to remain undisclosed. The data are not publically available, but were utilized in previous works to model users’ browsing behaviors (see for example, [11]). Despite this limitation, we make the anonymized network, together with the labels of all filtered users, available in a public repository [25].

We took several actions to ensure the data’s credibility. First, we only included users with a total number of URLs between 100 and 9000 over the entire three-month period to model a typical human browsing activity. Second, to ensure a continuous analysis, we included users whose first and last browsing sessions were at least 30 days apart. Altogether, our study includes about 25,000 qualified users.

### Data labeling

The Google Safe Browsing (GSB) API is a blacklist-based API that is often used for classifying URLs [26]. We consider a user to be *exposed* if she entered a URL that GSB classified as malicious. Correspondingly, we define users as *risky* in a specific learning period if they entered at least one malicious URL, and *safe* otherwise. By definition, all users are initially categorized as *safe* and can transition to the *risky* category, yet they cannot become safe again. Following 30, 60, and 90 days, 8.5%, 14.9%, and 16.35% of the users are considered risky. These percentages are within the range stated by other papers as the proportion of risky users in society [3, 4]. Note that the classification is URL-specific and not domain-specific. Namely, the percentage of URLs that are considered malicious in a given domain is often low, and most domains containing malicious URLs also have many completely safe ones.

### Splitting the data

Our goal is to predict which users will enter a malicious URL within 60–89 days by studying the users’ behavior during the first 0–59 days. These full 90 days were the same calendric days for all users. We chose the same calendric days to ensure no data leakage to the test set. We randomly split the data (i.e., the users, their features in every feature set, and their labels), using 80% of the data for training and the rest for test purposes. We perform 10-fold cross-validation on the train set itself, where 90% of the users are used for learning and the other 10% for validation in every iteration. The performances of the final classifiers are tested on 20% of the users who were not used in training and unseen by any of the models.

### Feature extraction for Network Framework

The Network Framework distinguishes between safe users, UCRUs and CRUs. For safe users, it predicts that their probability of becoming infected is 0. For UCRUs, we use the URR as a feature for prediction. For CRUs, we use features we extracted from the network using the node2vec algorithm [27]. This method allows learning continuous feature representations for nodes in networks. It takes a graph of the form *G*(*V,E,W*) as input and treats every node as a ‘word’. It then simulates random walks between nodes in the graph to create ‘sentences’ that add up to a ‘corpus’, similar to the textual corpora used in natural language processing models. The output of the algorithm is an embedding (a vector representation) for each of the graph’s nodes. We define the size of the output vector to be 64, in order to balance between the number of features and the total information each one ‘represents’. After running the algorithm on the graph, similar nodes are represented by similar vectors. Due to the structure of the algorithm, similar nodes are considered as such if they play similar roles in their connected component and in the entire network.

To incorporate the second layer in our predictions for CRUs, we combine the extracted node2vec features with the URR.

To incorporate the third layer, we depict two traits for every user comprised of binary vectors of size 60, corresponding to the number of days in the learning period. The first indicates the day on which each user joined the CRUN. The second indicates the days following the joining day in which the user entered at least one malicious URL (Fig 2).

### The Browsing Framework–baseline comparison

We compare our framework to a *Browsing Framework baseline*, which includes features previously shown to be effective for malware prediction [3, 4]. Specifically, for each user, we extract a total of 47 features. Each feature belongs to one of three groups: general browsing habits, risky browsing habits, and time-related habits. The *general browsing habits* group includes features such as the total number of sessions, the user’s average session duration, and her number of URLs per session. The *risky browsing habits* group includes similar features, specifically regarding sessions that contained a malicious URL. An example of such a feature is the total number of sessions in which the user entered at least one malicious URL. *The time-related habits* group portrays the hours of the day during which the user was most active (for the complete list of features see S2 Text. Feature extraction for the browsing framework).

To ensure a complete and fair comparison of results on the CRUs population, the Browsing Framework contains two different classifiers, which differ from one another according to the population on which they were trained. The *exclusive* Browsing classifier is trained only on CRUs. The *inclusive* Browsing classifier is trained on the entire population. We compare the latter’s results both on the whole population and on the CRUs group alone.

Overall, the standard browsing framework includes various types of information on risky and safe web surfing. It aims to capture the broad picture of the user’s browsing behavior to predict upcoming infections. Thus, it serves us as a comparison baseline for our model’s predictions and the levels of personal user-information required.

### Defining the classifiers

We compare the results of our models on three separate groups: the CRUs, the risky population (CRUs and UCRUs), and the entire population. In this section, we describe how the frameworks are tested against one another under the same conditions; where relevant, we tuned and optimized the classifiers’ hyper-parameters to achieve the best results possible on the training set. All models were trained/tested on the same train/test populations, accordingly. We also run a feature importance process to filter out less-informative features prior to the training of every model. This step is conducted with an initial Random Forest algorithm that was run prior to the training of the model, using the minimal entropy split criterion. For the Network and Browsing Frameworks, we used the following classifiers: decision trees, logistic regression, artificial neural networks, Random Forests, and XGBoost, intending to maximize the area under the receiver operating characteristic curve (AUC). The training process included a hyper-parameter tuning step prior to the fitting of the model, in order to ensure its robustness and preciseness. This step was done in a grid-search approach, testing out different combinations of values for each hyperparameter of each model and choosing the best performing set of values. In all of the scenarios, the Random Forest algorithm outperformed its alternatives in the training step during the cross-validation step, and thus we present its results on the test set. After the final models are chosen, a feature importance analysis is conducted to better understand which features had the most effect on the predictive power of each model (see Fig in S2 Text. Feature extraction for the browsing framework, and S1 Fig).

## Results

### First layer classifier

**Fig 3:**
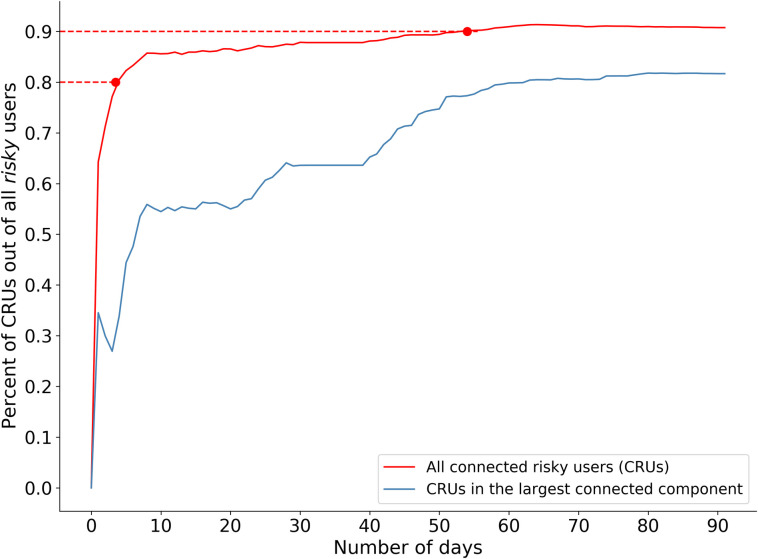
Connected Risky User Network. The percentage of risky users who belong to the CRUN grows larger over time (shown in red). By the end of the 4^th^ day, 80% of the risky users belonged to it. By the end of the 54^th^ day, 90% of them did. We observe similar patterns in the growth over time of the network’s largest connected component (shown in blue).

**Fig 4:**
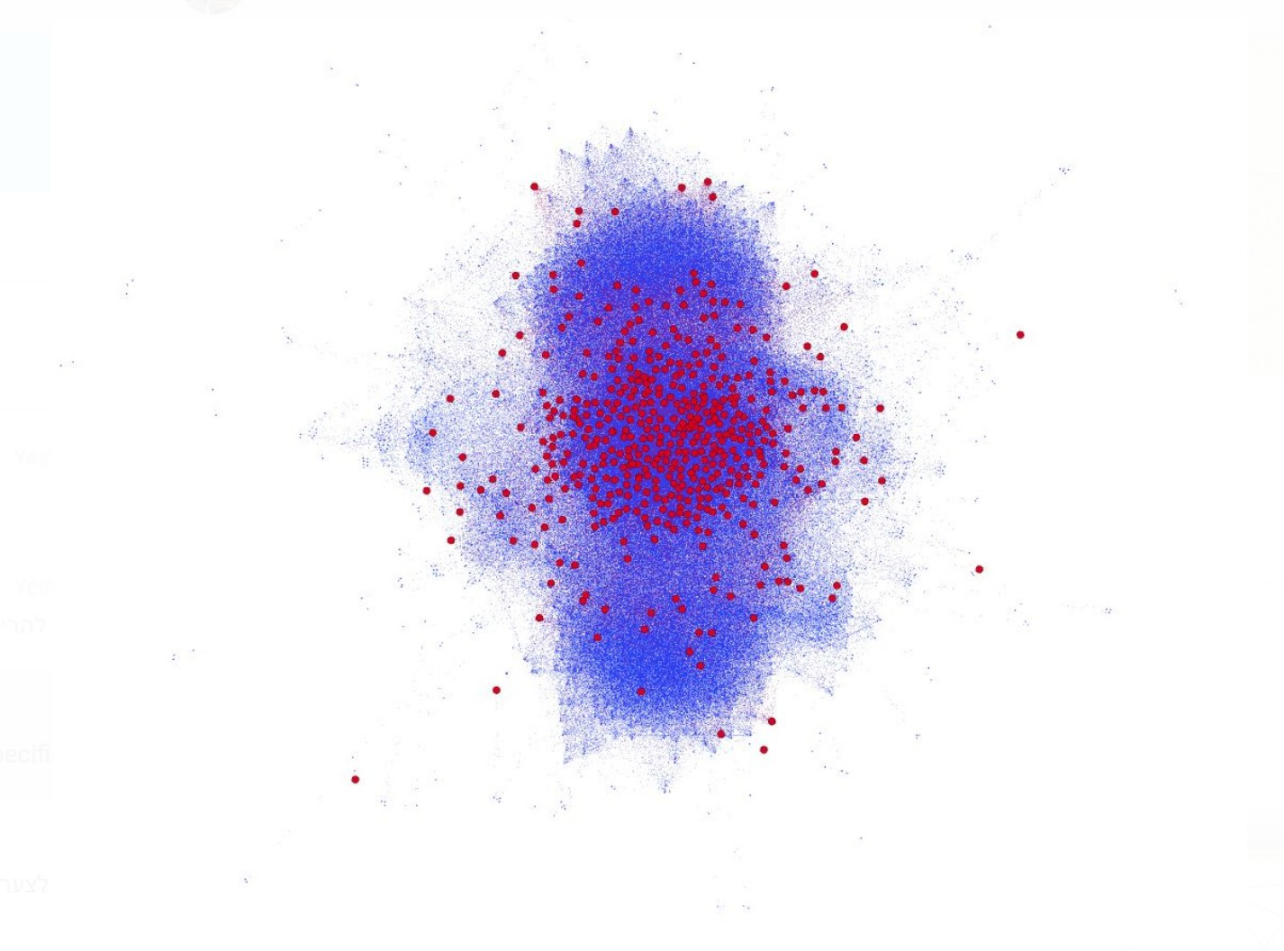
A representation of the CRUN. A representation during the learning (0–59 days) and test (60–89 days) periods. Nodes are colored red if they were exposed during the prediction period, and blue otherwise. This depiction shows how nodes in the network’s central and denser areas by the end of the learning period had a higher chance of becoming exposed in the prediction period.

During the 60-day learning period, 1/1000 of the URLs visited by the users were classified as malicious by the GSB. 14.9% of the users were exposed to at least one malware. We found that by the end of the 54th day, 90% of all risky users belong to the CRUN, meaning they had at least one neighbor. Furthermore, by the end of the learning period, despite having 105 connected components, the network’s largest connected component comprised 85% of all CRUs and 76% of all risky users (Fig 3). We also observed a certain tendency of denser areas in our network to be at a higher risk for future exposure (Fig 4), indicating that the user’s similarity in exposure patterns can explain her future risk. Indeed, using a feature set based on the *node2vec* algorithm on the generated network, our model yielded an ROC AUC score of 72.8% on the test set.

### Second layer classifier

**Fig 5:**
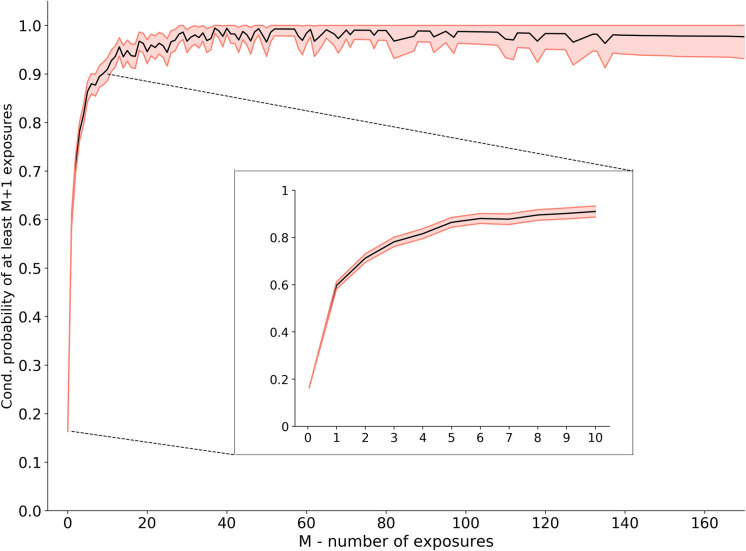
Risk rate given prior exposure. Given the group of users with at least *m* malware infections (X-axis), the value in the Y-axis represents the percentage of these users who became infected at least *m*+1 times. The more times a user becomes infected, the higher her probability of becoming infected again. The red outline represents a 95% confidence interval.

In the next step, we examine the contribution of the URR to the Network Framework’s prediction capability. Our initial tests on the data show that merely monitoring the number of exposures per user holds substantial information regarding the user’s tendency to become infected. Specifically, we observe that the higher the number of infections a user undergoes, the higher her probability of becoming infected again (Fig 5). We calculate each user’s URR feature, which considers only her total number of previous infections and total browsing time, and use it with the *node2vec* features as input for the second layer classifier. Utilizing the URR feature yields an addition of 6.4% to the area under the curve, leading to an ROC AUC score of 79.2%.

### Third layer classifier and model comparison

The user’s rate of entering risky URLs in the learning period and how the network evolves contributed to improving the prediction. Adding features that track the day in which a user joins the CRUN and the days of subsequent exposures after joining the CRUN improved the ROC AUC score to 80.9%. The ROC AUC scores for each framework are presented in Table 1.

**Table 1:** ROC AUC scores on the CRUs group in the prediction period.

*Framework (FW)*	*Layer / Version*	*CRUs*
*Network FW*	First layer classifier–*CRUN features*	72.8%
	Second layer classifier–*CRUN* and URR	79.2%
	**Third layer classifier**	**80.9%**
*Browsing FW*	Inclusive (trained over entire population)	76.7%
	Exclusive (trained over CRUs)	76.4%

**Fig 6:**
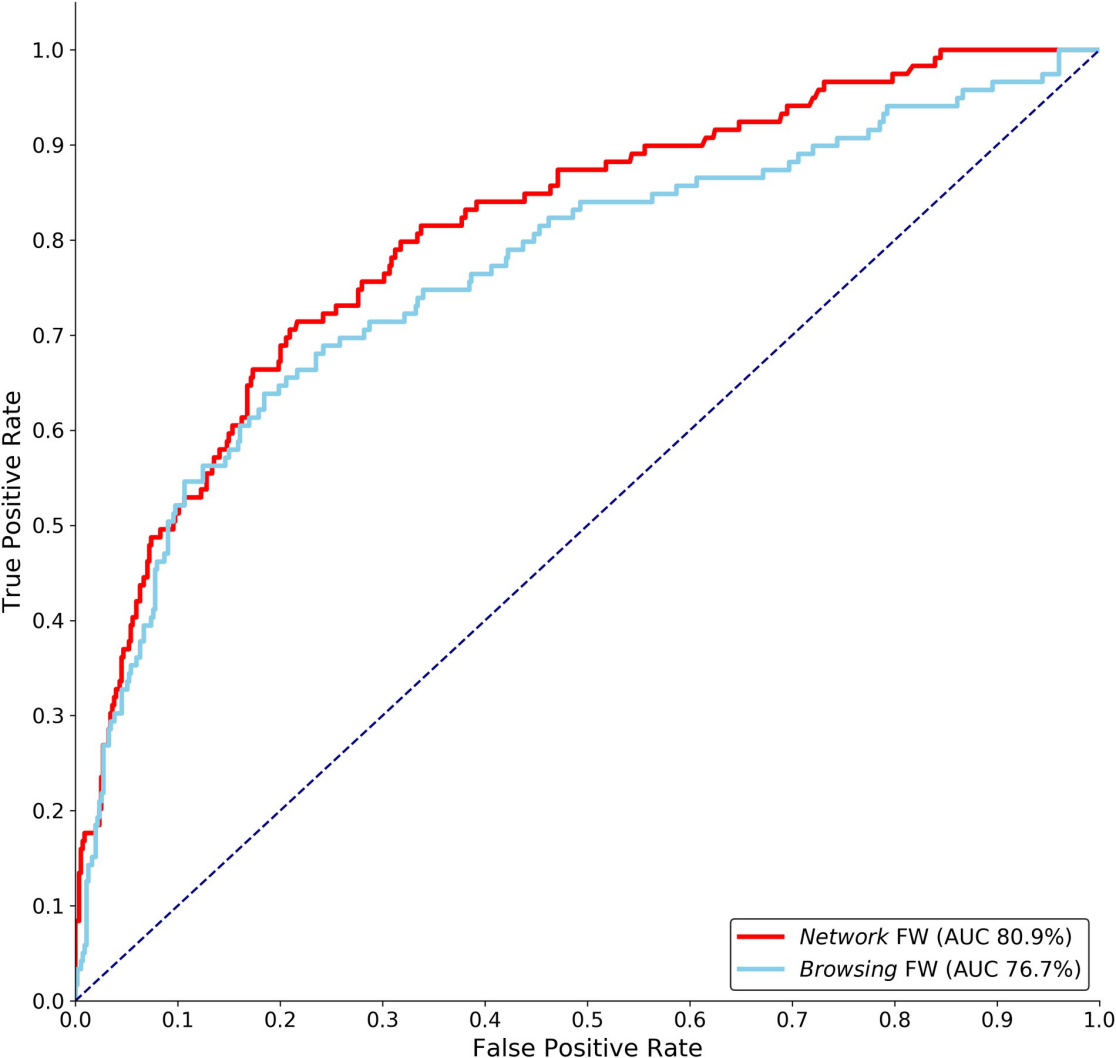
Receiver Operator Characteristic curves of the classifiers that perform best on the CRUs group for each framework. The red curve represents the Network Framework with all three exposure-related factors. The blue curve represents the *Inclusive* Browsing Framework, which was trained over the entire population of users. Regardless of the setting considered, the Network Framework outperforms the Browsing Frameworks.

To compare our methodological framework, we test two versions of the Browsing Framework–one that is trained over all types of users and one using the CRUs (i.e., only the users that belong to the Network Framework). We denote that the standard browsing framework considers a wide variety of information regarding the users’ browsing patterns, including session time, duration, and time-related habits. Regardless of the setting considered, the Network Framework achieves a higher score than the Browsing Frameworks (Table 1 and Fig 6).

## Discussion

The ability to apply cybersecurity means of protection to users without invading their privacy is crucial. Therefore, we showed a straightforward methodological framework that considers solely previous exposures to malicious content to improve malware detection. The Network Framework’s succeeded in predicting the CRUs population’s exposures, with a feature set derived only from their exposure-related activity. The most considerable contribution to the Network Framework’s prediction power lies in the features extracted from the CRUN. We found that the simple notion of a user-network, where connections are made based on similar exposure patterns, has a strong capability of assessing each user’s level of risk. Moreover, the ability to construct this network, based only on mutual domains and disregarding the URL content, highlights the strength of achieving these results with reduced privacy invasion.

The user risk rate (URR) impact is substantial, allowing the first layer classifier to attain better results than those of the best performing Browsing Framework. This feature also relies on two fundamental parts of the user’s infection-related activity: their total browsing time and their total number of past exposures. Interestingly, past infections as a predictor for future infections has been shown in other domains including infectious diseases [24].

Finally, the addition of the Spatio-temporal layer, which accounts for the patterns in which each user’s neighbors became infected during each day of the learning period, improves the results of the second layer classifier and outperforms the Browsing Framework.

When examining the frameworks’ performance on the entire risky population, the Network Framework’s results exceed those of the Browsing Framework, but with a slightly smaller margin (3.3%). The drop in the margin could be related to the fact that the predictions of about 10% of the risky users are based only on their URR values, since they do not belong to the CRUN and therefore do not benefit from its strong features. Even so, results suggest that once a user transitioned to the *risky* class, the Network Framework serves as a better predictor for assessing her level of risk.

When considering the entire population, the Browsing Framework’s score is better than that of the Network Framework, with the difference being 3.6%. These results are not surprising as the Browsing Framework is trained upon the entire population and can predict that a safe user will become infected in the prediction period. The Network Framework, in its suggested configuration, has the limit of not incorporating information of the safe users. One possible approach to address this matter is to define a spectrum of riskiness for each URL (instead of a strict binary categorization), and then try to identify relatively subtle riskiness-tendencies amongst safe users. This approach requires an API that either offers a soft categorization for each website, or combining multiple APIs and setting the score of each URL to be the average label across all APIs. Due to the millions of URLs that required labeling for our analysis, we were limited to specific APIs for which we had enough labeling quota, and these enhancements were left outside the scope of this work.

The Browsing Framework also shows somewhat less powerful results than similar models introduced in other studies [4, 7]. This can be accounted for our data’s ’impurity’ compared to theirs. Specifically, the data we analyzed is limited to personally-installed browser software versus data collected from the users’ cellular service provider or data collected from a popular AV). Another possible explanation would be that we did not refer to the categories of the users’ websites, which may hold valuable information regarding the users’ level of riskiness (e.g., adult-content websites tend to contain more malware than news websites [4, 7]). As these website categorizations were not applied in the Network Framework, we assume both frameworks’ scores are similarly affected by the absence of these features.

The notions portrayed in the Network Framework can be applied in real-life systems following slight modifications, and allowing effective and non-invasive user-level risk-assessment. A system incorporating these features as part of its security measures could dynamically alter each user’s estimated risk of exposure, efficiently distribute resources and attention, and avoid creating harsh limitations where they are not required. In this sense, the framework in itself is not a real-time system but a periodic one; in order to incorporate it, a training and assessment process should be conducted frequently to reevaluate each user’s level of risk for the upcoming period. However, given enough data, our framework strongly suggests that risky users can be recognized very early, and that these categorizations hold for a reasonable amount of time. In a real life system with corresponding resources, we believe that the reevaluation process could be held more frequently, such as every week or day. The window-to-the-past should be adjusted accordingly, but the results suggest that two-months of historic data should suffice both for the construction of the network and for the evaluation of the URR feature. This reassessment approach would assist in capturing the risk level of users that change their browsing behavior from risky to safe and vice-versa over time.

## Conclusion

We present a novel personalized framework for predicting user-level entrances to malicious webpages. We show how these predictions are feasible even when we analyze partial data from the user’s exposure patterns, which corresponds to less than a thousandth of the URLs the user enters. Our framework indirectly accounts for the user’s most basic risky browsing behavior by identifying her similarity in exposure patterns to other users. Specifically, we portray how only tracking the domains in which users had entered malicious URLs and the users’ total browsing time, and their total number of past exposures, can suffice as features for our prediction models. This approach allows a significant reduction of privacy invasion. Overall, this concept may serve security systems as an additional layer, focusing on user behavior, which is relatively straightforward to predict, instead of the growingly sophisticated malware behavior.

## Supporting information

S1 Fig Feature importance for the Network Framework. In the tuning and validation process of the Network Framework, the Random Forest algorithm outperformed its alternatives, with the split criterion chosen to be entropy. Since the node2vec features are essentially embeddings, examining each bit separately does not provide much information. Instead, we present in this plot the cumulative importance of feature groups based on the number of the layer. The first layer classifier only uses the node2vec features. In the second layer classifier, the URR is added, accounting for nearly 20% of the reduction in entropy throughout the splits of the algorithm. The combined contribution of the node2vec and the URR add up to 69.5% in the third layer classifier, with the addition of the Spatio-temporal features accounting for just over 30%.
(PDF)

S1 Gif (GIF)

S1 Text Statistical analysis for the URR. (PDF)

S2 Text Feature extraction for the Browsing Framework. (PDF)
