# Supplementary material for: Co-similar malware infection patterns as a predictor of future risk
Source: PLoS One. 2021 Mar 29;16(3):e0249273. doi: 10.1371/journal.pone.0249273 (PMC8007008; doi:10.1371/journal.pone.0249273)
Supplement: S1 Fig — In the tuning and validation process of the Network Framework, the Random Forest algorithm outperformed its alternatives, with the split criterion chosen to be entropy. Since the node2vec features are essentially embeddings, examining each bit separately does not provide much information. Instead, we present in this plot the cumulative importance of feature groups based on the number of the layer. The first layer classifier only uses the node2vec features. In the second layer classifier, the URR is added, accounting for nearly 20% of the reduction in entropy throughout the splits of the algorithm. The combined contribution of the node2vec and the URR add up to 69.5% in the third layer classifier, with the addition of the Spatio-temporal features accounting for just over 30%. (PDF) [file pone.0249273.s001.pdf]

Supporting information: Co-similar Malware Infection Patterns as a Predictor of Future Risk

Amir Yavneh[1], Roy Lothan[1], Dan Yamin[1*]

1 Department of Industrial Engineering, Faculty of Engineering, Tel Aviv University,

Tel Aviv 69978

* Correspondence to Dan Yamin dan.yamin@gmail.com

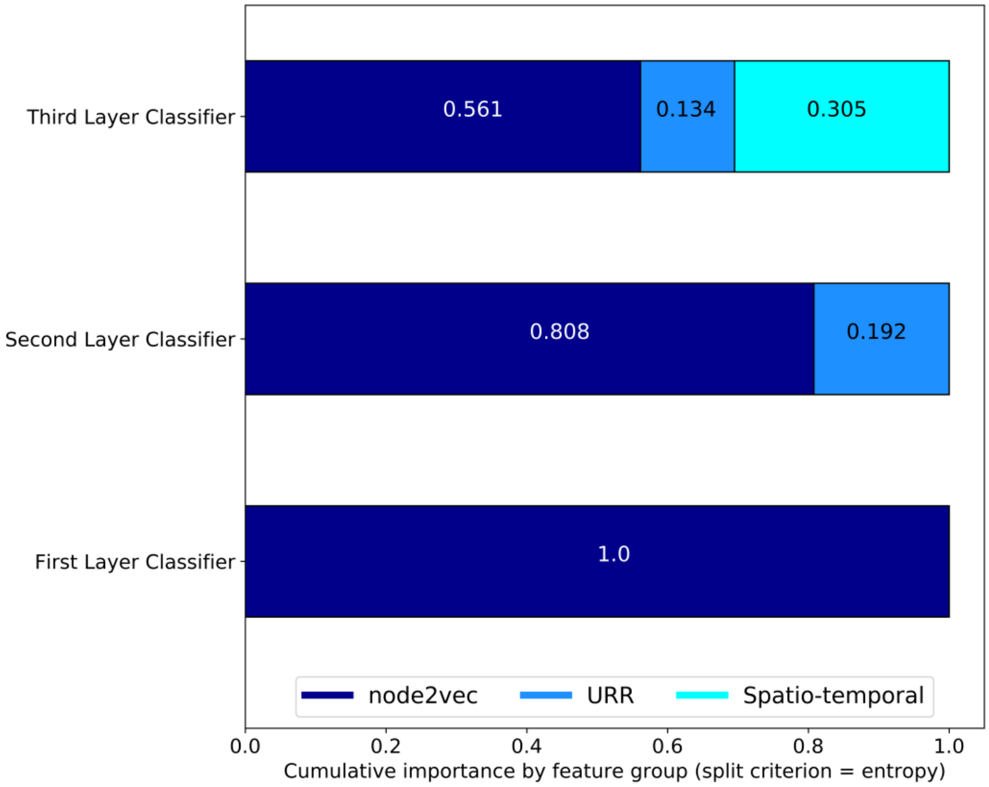

S3 Fig. Feature importance for the Network Framework In the tuning and validation process of the Network Framework, the Random Forest algorithm outperformed its alternatives, with the split criterion chosen to be entropy. Since the node2vec features are essentially embeddings, examining each bit separately does not provide much information. Instead, we present in this plot the cumulative importance of feature groups based on the number of the layer. The first layer classifier only uses the node2vec features. In the second layer classifier, the URR is added, accounting for nearly 20% of the reduction in entropy throughout the splits of the algorithm. The combined contribution of the node2vec and the URR add up to 69.5% in the third layer classifier, with the addition of the Spatio-temporal features accounting for just over 30%.
