# Supplementary figures and images for: Co-similar malware infection patterns as a predictor of future risk
Source: PLoS One. 2021 Mar 29;16(3):e0249273. doi: 10.1371/journal.pone.0249273 (PMC8007008; doi:10.1371/journal.pone.0249273)

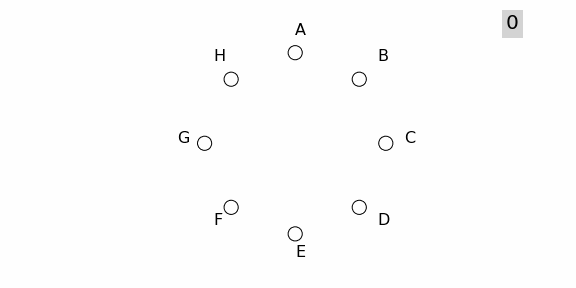

Supplement: S1 Gif — (GIF) [file pone.0249273.s002.GIF]
