# Supplementary material for: Co-similar malware infection patterns as a predictor of future risk
Source: PLoS One. 2021 Mar 29;16(3):e0249273. doi: 10.1371/journal.pone.0249273 (PMC8007008; doi:10.1371/journal.pone.0249273)
Supplement: S1 Text — (PDF) [file pone.0249273.s003.pdf]

Supporting information: Co-similar Malware Infection Patterns as a Predictor of Future Risk

Amir Yavneh[1], Roy Lothan[1], Dan Yamin[1*]

1 Department of Industrial Engineering, Faculty of Engineering, Tel Aviv University,

Tel Aviv 69978

* Correspondence to Dan Yamin dan.yamin@gmail.com

S1 Text. Statistical analysis for the URR

Examining which part of the risky population has a statistically significant non-Poisson rate of entering malicious websites was done using the Lilliefors test [1]. The test is based on the Kolmogorov-Smirnov test, with the main difference being that it can be performed even when the parameters of the distribution are estimated from the data and are not priorly known, as with λ_u in our case. The actual test examines if the times between events – i.e., browsing duration between one entrance to a malicious URL to the next – follow an exponential distribution (H_0) or not (H_1). For this test, we considered all users with at least three exposures, which matched the minimum requirement to conduct the test (using the statsmodels [2] package in Python). Overall, only 27.2% of all risky users had statistically significant non-Poisson rates (p-value < 0.01).

[1] H. W. Lilliefors, "On the Kolmogorov-Smirnov Test for the Exponential Distribution with Mean Unknown," *J. Am. Stat. Assoc.*, vol. 64, no. 325, pp. 387–389, 1969.

[2] S. Seabold and J. Perktold, "Statsmodels: Econometric and Statistical Modeling with Python," 2010.
