# Supplementary material for: Co-similar malware infection patterns as a predictor of future risk
Source: PLoS One. 2021 Mar 29;16(3):e0249273. doi: 10.1371/journal.pone.0249273 (PMC8007008; doi:10.1371/journal.pone.0249273)
Supplement: S2 Text — (PDF) [file pone.0249273.s004.pdf]

Supporting information: Co-similar Malware Infection Patterns as a Predictor of Future Risk

Amir Yavneh[1], Roy Lothan[1], Dan Yamin[1*]

1 Department of Industrial Engineering, Faculty of Engineering, Tel Aviv University,

Tel Aviv 69978

* Correspondence to Dan Yamin dan.yamin@gmail.com

S2 Text. Feature extraction for the browsing framework

We extracted the following features as part of the Browsing Framework. Specifically, we divided the features into three categories: 1) general browsing habits, 2) risky browsing habits, and 3) time-related habits. All aggregations refer to the user's activity during the relevant learning period.

2.1 General Browsing Habits

1. The total number of sessions conducted by the user
2. The average session duration
3. The average session duration, disregarding sessions with a single URL
4. The standard deviation of all session durations
5. The standard deviation of all session durations, disregarding sessions with a single URL
6. The median session duration
7. The median session duration, disregarding sessions with a single URL
8. The total number of URLs the user entered
9. The total number of distinct domains the users entered
10. The average number of domains entered in a session

2.2 Risky Browsing Habits

11. The total number of sessions with at least one malicious URL (risky sessions)
12. The total number of sessions with at least one malware URL
13. The total number of sessions with at least one social-engineering URL

14. The percent of sessions in which the user entered at least one malicious URL
15. The percent of sessions in which the user entered at least one malware URL
16. The percent of sessions in which the user entered at least one social-engineering URL
17. The average session duration for risky sessions
18. The median session duration for risky sessions
19. The average session duration for risky sessions, disregarding ones with a single URL
20. The standard deviation of all risky session durations
21. The median session duration for risky sessions, disregarding ones with a single URL
22. The total number of malicious URLs the user entered - consists of 'malware', 'social engineering', and 'Google unwanted shavar' URLs
23. The total number of malware URLs the user entered
24. The total number of social-engineering URLs the user entered
25. The total number of Google unwanted shavar URLs the user entered
26. Percent of malware URLs out of all malicious URLs
27. Percent of social-engineering URLs out of all malicious URLs
28. Percent of Google unwanted-shavar URLs out of all malicious URLs
29. Percent of malware URLs out of all URLs
30. Percent of social-engineering URLs out of all URLs
31. Percent of Google unwanted-shavar URLs out of all URLs
32. The total number of distinct domains the user entered, only regarding sessions with at least one malicious URL
33. The total number of domains in which the user entered a malicious URL
34. The total number of distinct domains in which the user entered a malicious URL, divided by the number of distinct domains the user visited
35. Average number of domains in a session where a malicious URL was entered

36. On average - per bad session, how early was the first malicious URL entered (i.e., in terms of the order, not the time)
37. How early was the first session that the user got infected (first risky session out of all risky sessions)
38. Average number of URLs visited between two malicious URLs

2.3 Time-related Habits

39. The total number of URLs entered during 5:00-8:00
40. The total number of URLs entered during 8:00-11:00
41. The total number of URLs entered during 11:00-12:00
42. The total number of URLs entered during 12:00-15:00
43. The total number of URLs entered during 15:00-17:00
44. The total number of URLs entered during 17:00-19:00
45. The total number of URLs entered during 19:00-21:00
46. The total number of URLs entered during 21:00-23:00
47. The total number of URLs entered during 23:00-05:00

All time-dependent features are in terms of the user's local time. The original data was given in UNIX time; to find the time of day in the user's time zone, we used the country id to extract the time zone (if the country id was missing, the default time zone used was Greenwich). Using the UNIX time and the time zone, we converted the timestamp to the user's local time.

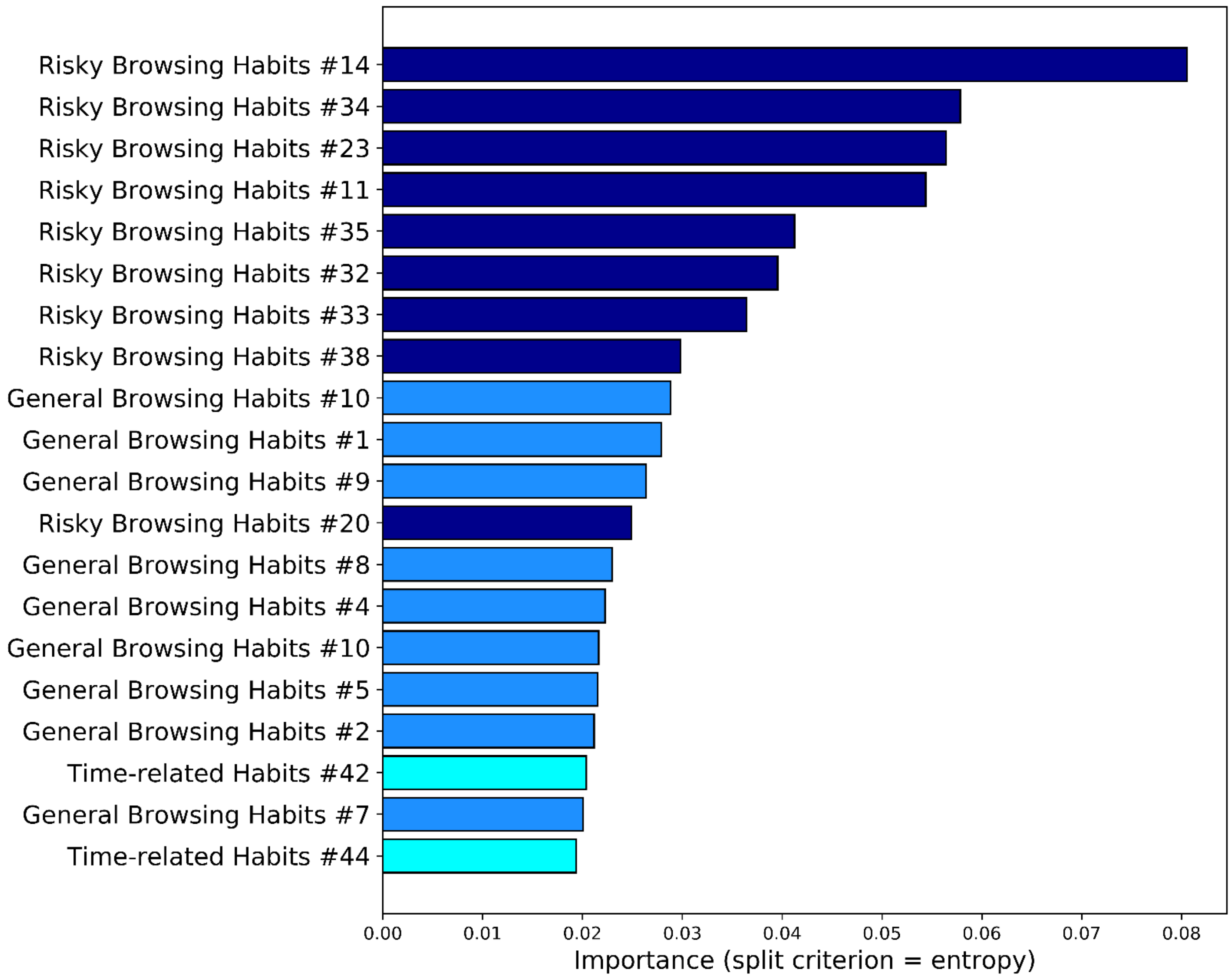

S2 Fig. Feature importance analysis for the Browsing Framework In the tuning and validation process of the Browsing Framework, the Random Forest algorithm outperformed its alternatives, with the split criterion chosen to be entropy. The top 20 most important features on average are presented in this plot. As shown, the majority of the most important features are related to the risky behavior of the user. The numbers of the features refer to the list provided in *S2 Text Feature extraction for the browsing framework.*
